# The SMAC Mimetic APG-1387 Sensitizes Immune-Mediated Cell Apoptosis in Hepatocellular Carcinoma

**DOI:** 10.3389/fphar.2018.01298

**Published:** 2018-11-06

**Authors:** Zide Chen, Jiehua Chen, Hongyan Liu, Wei Dong, Xuan Huang, Dajun Yang, Jinlin Hou, Xiaoyong Zhang

**Affiliations:** ^1^State Key Laboratory of Organ Failure Research, Guangdong Provincial Key Laboratory of Viral Hepatitis Research, Department of Infectious Diseases, Nanfang Hospital, Southern Medical University, Guangzhou, China; ^2^Hepatic Surgery Center, Tongji Hospital, Tongji Medical College, Huazhong University of Science and Technology, Wuhan, China; ^3^Department of Medical Oncology, Sun Yat-sen University Cancer Center, Guangzhou, China; ^4^Ascentage Pharma Group Corporation Limited, Suzhou, China

**Keywords:** SMAC mimetic, inhibitor of apoptosis protein, TNF-α, TRAIL, natural killer cells, cancer stem cells

## Abstract

The inhibitor of apoptosis protein (*IAP*) genes are frequently overexpressed in malignancies. Second mitochondria-derived activator of caspase (SMAC) mimetics, which target IAPs, have potential to trigger cancer cell death and sensitize tumor cells to cytotoxic therapy. The aim of this study was to investigate the anti-tumor potential of a novel bivalent SMAC mimetic, APG-1387, in hepatocellular carcinoma (HCC). The mRNA and protein expressions of IAPs, including cellular IAPs (cIAP1 and cIAP2) and X chromosome-linked IAP (XIAP), were increased in HCC tumors compared with normal liver tissue. APG-1387 treatment alone significantly reduced the protein levels of IAPs, but had only a modest effect on the viability and apoptosis of HCC cells *in vitro*. However, APG-1387 in combination with tumor necrosis factor-alpha (TNF-α) or tumor necrosis factor-related apoptosis-inducing ligand (TRAIL) significantly reduced cell viability and proliferation, and induced apoptosis in HepG2 cells, as well as in HCCLM3 cells that harbors cancer stem cell-like properties. These synergistic killing effects were caspase-dependent and partially dependent on RIPK1 kinase activity. Furthermore, APG-1387 also promoted the killing effect of Natural Killer cells on HCC cells *in vitro* and the combination therapy significantly inhibited tumor growth by inducing cell apoptosis in xenograft mice model. In conclusion, our study clarified that APG-1387 could sensitize HCC cells to cytokines or immune cells mediated cell killing and implied that potential of SMAC mimetic based combination immunotherapy for HCC treatment.

## Introduction

Worldwide, hepatocellular carcinoma (HCC) is the third leading cause of cancer-related mortality, with increasing incidence and poor prognosis (Ferlay et al., 2015). Liver transplantation, liver resection and liver tumor ablation offer potential curative options for patients with early-stage HCC, but there are still a large number of tumor recurrences and metastases. In patients with intermediate- and advanced-stage HCC, only palliative treatments exist, and these include transarterial chemo-embolization, chemotherapy, and anti-angiogenic therapy, all of which have limited efficacy (Forner et al., 2012). Therefore, there remains a need for the development of novel therapeutic strategies to improve the prognosis for patients diagnosed with HCC.

Apoptosis resistance represents one of the hallmarks of human cancer and is a major reason why many anti-cancer treatment regimens fail (Hanahan and Weinberg, 2011). Inhibitor of apoptosis proteins (IAPs), including cellular IAP1 (cIAP1), cellular IAP2 (cIAP2) and X chromosome-linked inhibitor of apoptosis protein (XIAP), are a family of anti-apoptotic proteins that block the caspases activation cascade (Fulda and Vucic, 2012). XIAP directly binds to and inhibits caspase-3, -7, and -9, while cIAP1 and cIAP2 prevent cell death-receptor complex formation and caspase-8 activation (Fulda and Vucic, 2012). It has been known that IAPs are frequently abnormally upregulated in human malignancies, including HCC (Fulda and Vucic, 2012). Also, genetic data have shown that the genome region that carries *cIAP1* and *cIAP2* is amplified in HCC and other tumor types (Dai et al., 2003; Zender et al., 2006). Therefore, IAPs might have potential as therapeutic targets in HCC therapy.

The second mitochondria-derived activator of caspase (SMAC) protein is an endogenous IAP antagonist released from mitochondria during cell apoptosis and can promote apoptosis by removing IAP inhibition of caspases (Martinez-Ruiz et al., 2008). Small molecules that mimic the binding interaction between IAPs and SMAC, termed ‘SMAC mimetics,’ can inhibit the expression of IAPs, resulting in caspase activation (Fulda, 2015a). In HCC, the expression of SMAC protein has been shown to be down-regulated in tumor tissue compared with normal adjacent liver tissue (Okano et al., 2003). Also, treatment with SMAC mimetics (Tian et al., 2014; Liese et al., 2015), silencing IAPs with small interfering RNAs (siRNAs), or exogenously increasing SMAC expression, have been shown to facilitate apoptosis of HCC cells in response to chemotherapy or cytokine treatment (Okano et al., 2003; Yamaguchi et al., 2005; Chen et al., 2006; Liu et al., 2010; Li et al., 2013).

Currently, several SMAC mimetics have been designed and are undergoing evaluation in early clinical trials as potential cancer therapeutic agents (Fulda and Vucic, 2012; Fulda, 2015a). APG-1387 is a novel bivalent SMAC mimetic that has been shown to have significant antitumor activities in ovarian cancer (Li et al., 2018), nasopharyngeal carcinoma (Li et al., 2016) and HBV-positive HCC cell line PLC/PRF/5 (Pan et al., 2018), but has yet to be evaluated in other HCC cell types that resistant to its monotherapy. In this study, we examined the expression of IAPs in human liver tumor tissues and investigated the combinational anti-tumor potential of APG-1387 with cytokines or immune cells in HCC cell lines that resistant to APG-1387 monotherapy, and in a mouse xenograft model of HCC.

## Materials and Methods

### Ethical Approval and Patient Consents

The study protocol conformed to the Helsinki Declaration of 1975 and it was approved by the Human Ethics Committee of Tongji Hospital and by the Ethics Committee of Nanfang Hospital. All human study participants provided written informed consent to participate in the study and to provide tissue and blood samples.

### Hepatocellular Carcinoma (HCC) Clinical Samples

Twelve patients with HCC who underwent tumor resection were randomly selected. Paired samples of HCC tissue and normal adjacent liver tissue were collected from Tongji Hospital, Tongji Medical College, Wuhan, Peoples’ Republic of China, between September 4th, 2012 and November 20th, 2013. The clinical data for the patients in the study are shown in Supplementary Table 1.

### Cell Lines and Reagents

The human HCC cell lines HepG2, HCCLM3, and Huh7 were obtained from the Cell Bank of Type Culture Collection (Chinese Academy of Sciences, Shanghai, China). These cells were cultured in DMEM medium (Thermo Fisher Scientific, Waltham, MA, United States) supplemented with 10% fetal bovine serum and 1% penicillin/streptomycin (Biological Industries, Kibbutz Beit Haemek, Israel) in a humidified incubator containing 5% CO_2_ in air at 37°C.

The APG-1387 compound was kindly provided by Ascentage Pharma Group Corp. Ltd. For the *in vitro* studies, APG-1387 was dissolved in sterile water at a concentration of 20 mM, kept at 4°C as a stock solution, and diluted to the required concentrations before use. For the *in vivo* experiments, APG-1387 was dissolved in 9% NaCl sterile water at a concentration of 2 μg/μl. Recombinant human TNF-α, TRAIL, interleukin (IL)-12, and IL-15 were purchased from PeproTech (Rocky Hill, CT, United States). Recombinant human IL-18 was purchased from Invivogen (San Diego, CA, United States). Verapamil HCl, the pan-caspase inhibitor Z-VAD-FMK, and necrostatin-1 were purchased from SelleckChem (Houston, TX, United States). Antibodies obtained from Cell Signaling Technology (Danvers, MA, United States) included anti-cIAP1 (cat. no. 7065), anti-XIAP (cat. no. 2045), anti-PARP (cat. no. 9532), anti-caspase 3 (cat. no. 9662), anti-cleaved caspase 9 (cat. no. 7237), anti-NIK (cat. no. 4994), and anti-β-actin (cat. no. 4967). The validation of cIAP1 and cIAP2 antibodies was available in Supplementary Figure 10. The following antibodies were obtained from Abcam (Cambridge, MA, United States): anti-cIAP2 (cat. no. ab32059), anti-GSDME (cat. no. ab215191) and anti-Sox2 (cat. no. ab137385). Anti-cleaved-caspase 8 (cat. no. 40502) was obtained from Signalway Antibody LLC (College Park, MD, United States).

### Quantitative Reverse Transcription Polymerase Chain Reaction (qRT-PCR)

Quantitative reverse transcription polymerase chain reaction (qRT-PCR) was performed, as previously described (Ge et al., 2017).Briefly, total RNA was isolated from HepG2, HCCLM3, or sorted cells using NucleoSpin RNA II (Macherey-Nagel, Duren, Germany) followed by DNase I treatment. After transcribing into cDNA using a Transcriptor cDNA Synth Kit (Roche, Basel, Swiss), the cycles of threshold (Ct) were detected by running real-time PCR in the Roche LighCycler 480 system using a miScript SYBR Green PCR kit (Qiagen, Hilden, Germany). The forward and reverse primers to detect mRNA were *cIAP1*(Qiagen, QT00083587); *cIAP2* (Qiagen, QT00021798); *XIAP* (Qiagen, QT00042854); *ABCG2*, AGGTCTGGATAAAGTGGCA and GAGGCTGATGAATGGAGAA; *CD44*, CTGCCGCTTTGCAGGTGTA and CATTGTGGGCAAGGTGCTATT; *SOX2* TACAGCATGTCCTACTCGCAG and GAGGAAGAGGTAACCACAGGG; and *β-actin*, TCCCTGGAGAAGAGCTACGA and AGCACTGTGTTGGCGTACAG. All samples were normalized to an internal control (β-actin), and the expression of each gene was presented as fold changes, which were calculated through relative quantification using the method 2^-ΔΔCt^.

### Western Blot

For the Western blot analysis, the total cellular protein of HCC cells was obtained by lysing the cells with RIPA lysis buffer. Protein concentrations were determined using the bicinchoninic acid (BCA) assay (Fdbio Science, Hangzhou, China). The protein samples were mixed with 5× loading buffer, and heated for 10 min at 95°C. Samples were loaded to sodium dodecyl sulfate-polyacrylamide gel electrophoresis (SDS–PAGE) and transferred to a nitrocellulose filter membrane (GE Healthcare, Chicago, IL, United States), followed by incubation with the blocking buffer Tris-buffered saline/Tween 20 (TBST) in 5% bovine calf serum for 1 h. They were then incubated with primary antibody overnight at 4°C and horseradish peroxidase (HRP)-conjugated secondary antibody. The gray signal of the protein band was visualized with ECL Plus Western blotting detection reagents (GE Healthcare) using an ImageQuant LAS 4000 auto-exposure system (GE Healthcare), and semi-quantified by densitometry analysis.

### Cell Viability Using the Cell Counting Kit-8 (CCK-8) Assay

HepG2 and HCCLM3 cells were pre-inoculated in quadruplicate at 2,000 cells per well in 96-well plates for 12 h. After 24 h of stimulation with TNF-α or TRAIL, or in combination with APG-1387, the supernatants were replaced with 100 μl DMEM containing 10% CCK-8 (Dojindo, Kumamoto, Japan) solutions, and incubated at 37°C for 30 min and then optical density (OD) values were detected by a microplate reader (Bio Tek Gen5, Winooski, VT, United States) at a wavelength of 450 nm. The cell viability in the control group was defined as 100%, and the cell viability in other experimental groups was calculated accordingly.

### Flow Cytometry Analysis

After indicated treatments, all of the cells were collected separately, and incubated with Annexin-V and 7-amino actinomycin D (7-AAD) (BioLegend, San Diego, CA, United States) in the dark for 15 min. Then the apoptotic cells were detected on a FACSCanto^TM^ II Flow Cytometer (BD, Franklin Lakes, NJ, United States) by gating Annexin-V+/7AAD-, while necrotic cells were defined as Annexin-V+/7AAD+. Analysis of the side population (SP) by flow cytometry was performed, as previously described (Chiba et al., 2006). Specifically, HCCLM3 cells were detached from dishes using trypsin-EDTA (Gibco, Thermo Fisher Scientific, Waltham, MA, United States) and suspended at 1 × 10^6^ cells/ml in phosphate buffered saline (Life, Thermo Fisher Scientific, Waltham, MA, United States) supplemented with 2% fetal calf serum and 10 mM/L Hepes (Gibco, Thermo Fisher Scientific, Waltham, MA, United States). Then the cells were incubated with 20 μg/ml Hoechst 33342 (Sigma-Aldrich, St. Louis, MO, United States), either alone or in the presence of 500 μM verapamil (Selleckchem, Houston, TX, United States), at 37°C for 90 min in the dark with 15 min interval mixing. After incubation, the cells were washed twice with PBS, then 1 μg/ml prodium iodide (MP Biomedicals, Santa Ana, CA, United States) was added 5 min^TM^ before analyzing on the FACSAria^TM^ III Flow Cytometer (BD, Franklin Lakes, NJ, United States). Hoechst 33342 was activated with ultraviolet laser at 375 nm and fluorescence emission was measured with 450/BP40 (Hoechst blue) and 530/BP30 (Hoechst red) optical filters. Verapamil is used as a negative control for validation of SP cells. After adding of verapamil, gated SP cells failed to efflux Hoechst 33342 dye and stained with Hoechst 33342 positive. Both the SP and the remaining main population (MP) cells were sorted for qRT-PCR and cell viability analyses.

### Colony Formation Assay

HepG2 and HCCLM3 cells were pre-seeded in triplicate at 1,000 cells per well in 6-well plates for 12 h and then stimulated with 2 μM APG-1387, 100 ng/ml TNF-α, 20 ng/ml TRAIL or their combination. After incubation for 14 days, the colonies were fixed with 100% methanol for 2 min followed by staining with Giemsa dye for 30 min (Solarbio, Beijing, China), and counted with ImageJ software.

### Isolation of Natural Killer (NK) Cells and Co-culture

PBMCs from healthy donors were separated from whole blood on Lymphoprep^TM^ density gradients (Axis-Shield, Dundee, United Kingdom). CD56-positive NK cells were isolated from fresh PBMCs using CD56 MicroBeads (Miltenyi Biotech, Bergisch Gladbach, Germany), as previously described (Ge et al., 2017). The purity of the selected CD56-positive NK cells was >95%, as determined by flow cytometry using anti-α-CD3-APC-Cy7 (BD, Franklin Lakes, NJ, United States, cat. no. 557832) and anti-α-CD56-FITC antibodies (BioLegend, San Diego, CA, United States, cat. no. 318304). HepG2 or HCCLM3 cells were pre-seeded at a density of 5 × 10^5^ cells/well in 6-well plates for 12 h, and co-cultured with 2.5 × 10^5^ purified NK cells, with and without addition of 10 ng/ml IL-12, 10 ng/ml IL-15, and 100 ng/ml IL-18, and with or without the addition of 2 μM APG-1387. After co-incubation for 24 h, the supernatants were collected for lactate dehydrogenase (LDH) assay (Dojindo, Kumamoto, Japan) and enzyme-linked immunosorbent assay (ELISA) analysis (eBioscience, Thermo Fisher Scientific, Waltham, MA, United States) according to manufacturer instructions, while the cells were collected for flow cytometry analysis of apoptosis and necrosis.

### NK Cell Expansion and Xenograft Experiments

Natural killer cells were amplified using a human NK cell expansion kit (Dakewe, Beijing, China) in accordance with the protocol recommended by the manufacturer. All animal procedures were performed according to the Guide for the Care and Use of Laboratory Animals and approved by the Animal Care and Ethics Committee of Nanfang Hospital. A total of 20 male NOD-SCID mice (Charles River Laboratories, Wilmington, DE, United States) aged 4–6 weeks, were implanted with 5 × 10^6^ human HCC cell line HCCLM3 cells, subcutaneously into the right axillary cavity. Seven days later, when the tumors grew to 80–100 mm^3^ in size, the mice were randomized to four groups, with no significant difference in tumor size between the groups. The NOD-SCID mice were intraperitoneal injected with APG-1387 at 20 mg/kg every 3 days for 4 weeks, and/or injected at the peri-tumor site with 2 × 10^7^ IL-12, IL-15, and IL-18 activated NK cells per mouse every 6 days for 4 weeks. Same amount of phosphate-buffered saline (PBS) was used for injection control. Tumor volumes were calculated as length × width^2^/2 at indicated time points.

### Statistical Analysis

Before data analysis, the Shapiro–Wilk normality test was conducted to demonstrate a normal distribution of the data. Paired Student’s *t*-tests were used to compare the differences between the data from the HCC tissues and the adjacent normal liver tissues, and to compare the effects of the NK cell tumor cytotoxicity. Two-sample unpaired *t*-tests were conducted to assess the statistical significance of the changes in cytotoxicity, apoptosis, and colony numbers associated with TNF-α or TRAIL treatment with APG-1387 stimulation, gene expression, the percentage of SP cells, tumor volume, and tumor weight. All statistical analyses were performed using GraphPad Prism 6.01 (GraphPad Software Inc.).

## Results

### IAP Gene-Associated Proteins Were Highly Expressed in HCC Tumor Tissue Compared With Adjacent Normal Liver Tissue

**FIGURE 1:**
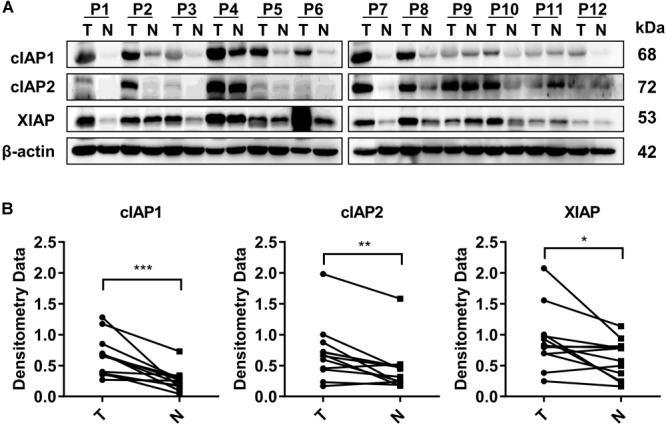
Proteins associated with the inhibitor of apoptosis protein (*IAP*) gene were highly expressed in HCC tissue. **(A)** The protein levels of cIAP1, cIAP2, and XIAP in 12 pairs of tissue samples of HCC were measured by Western blot. **(B)** The bands were semi-quantified by densitometry and normalized to β-actin. P, HCC patient; T, HCC tumor tissue; N, normal adjacent liver tissue; kDa, kilodalton. ^∗^*p* < 0.05, ^∗∗^*p* < 0.01, ^∗∗∗^*p* < 0.001, NS, not significant, by two-tailed pair *t*-test.

The expression of all three IAP-associated proteins, cIAP1, cIAP2, and XIAP, were increased in HCC tumor tissues compared with the normal adjacent liver tissues based on Western blot analysis of 12 hepatectomy samples. Increased expression of cIAP1 was present in 10/12 cases of HCC, increased expression of cIAP2 was present in 9/12 cases of HCC, and increased expression of XIAP was present in 9/12 cases of HCC (Figure 1A). The normalized densitometry of IAP protein bands on Western blot is shown in Figure 1B, which is consistent with previously reported findings (Tian et al., 2014). Immunohistochemistry analysis revealed that cIAP1 and cIAP2 were exclusively expressed in the cytoplasm of both tumor and non-tumor hepatocytes and were higher in tumor tissues than normal surrounding tissues (Supplementary Figure 1). To further confirm the increased expression of IAPs in HCC, The Cancer Genome Atlas (TCGA) database^1^ was reviewed, and RNA-seq data were collected from 49 paired HCC samples (Supplementary Figure 2a and Supplementary Table 2). Consistently, the fragments per kilobase of transcript per million mapped reads (FPKM) of RNA-seq data indicated that the mRNA levels of *cIAP1, cIAP2*, and *XIAP* were significantly lower in non-tumor tissue compared with tumor tissue (Supplementary Figure 2b). These results suggested that IAPs might act as oncogenes and be involved in the HCC development.

### APG-1387 Monotherapy Induced Rapid Degradation of cIAPs but Did Not Induce Cell Apoptosis in HepG2 and HCCLM3 Cells

**FIGURE 2:**
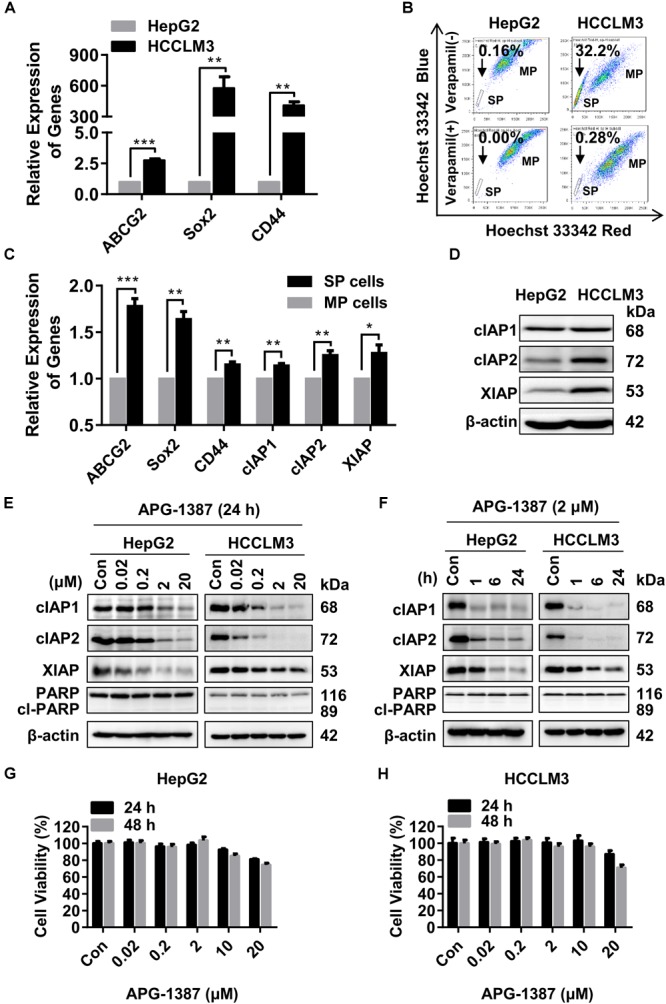
APG-1387 treatment induced rapid cIAPs degradation but failed to induce cell death in HepG2 and HCCLM3 cells. **(A,B)** The mRNA expression of cancer stem cell (CSC) genes (*ABCG2, CD44*, and *SOX2*) and the percentages of side population (SP) cells in HepG2 and HCCLM3 cells, were measured by qRT-PCR and flow cytometry, respectively. **(B)** The proportion of SP cells in HepG2 and HCCLM3 cell lines were 0.16% and 32.2%, which reduced to 0% and 0.28% in the presence of verapamil. **(C)** The relative expression of mRNA of CSC and *IAP* genes between SP and the main population (MP) HCCLM3 cells were examined by qRT-PCR. **(D)** The basic protein levels of cIAP1, cIAP2, and XIAP in HepG2 and HCCLM3 cells were analyzed by Western blot. **(E,F)** The changes in protein levels of the IAPs and cleaved poly (ADP-ribose) polymerase (PARP) proteins, **(G,H)** and cell viability of HepG2 and HCCLM3 cells were detected after treatment with AGP-1387 as a single agent at indicated concentrations and time periods. Con, control; cl-PARP, cleaved-PARP; kDa, kilodalton. Error bars represented the mean ± SEM of triplicate representative experiments; ^∗^*p* < 0.05, ^∗∗^*p* < 0.01, ^∗∗∗^*p* < 0.001, by two-tailed unpaired *t*-test.

It has previously been reported that low numbers of cancer stem cells (CSCs) are responsible for HCC metastasis and recurrence (Chiba et al., 2006). HCCLM3 is a human HCC cell line that harbors typical CSCs-like features, characterized by enriched SP cells (Guo et al., 2016). In the present study, qRT-PCR showed that genes linked to the CSC phenotype, including *ABCG2, SOX2*, and *CD44*, were expressed at much higher levels in HCCLM3 cells than HepG2 cells (Figure 2A). Also, the ratio of SP cells (based on Hoechst 33343 staining) in HCCLM3 was higher than that in HepG2 cells (Figure 2B). In addition, the expression levels of the *ABCG2, SOX2*, and *CD44* genes, as well as *cIAP1, cIAP2*, and *XIAP*, were increased in the SP cells compared to the other major population (MP) of cells (Figure 2C). Although cIAP2 and XIAP protein levels were higher in HCCLM3 than in HepG2 cells (Figure 2D), the expression of cIAP1 and cIAP2 in both cell lines decreased rapidly in a dose- and time-dependent manner following APG-1387 treatment, and the expression of XIAP was also inhibited by APG-1387 at a high dose (Figures 2E,F). Previously, it was reported that APG-1387 alone induced cell apoptosis in HBV positive PLC/PRF/5 cells (Pan et al., 2018), however, APG-1387 did not induce the expression of cleaved poly (ADP-ribose) polymerase (PARP), a marker for cell apoptosis in HepG2 and HCCLM3 cells (Figures 2E,F). This finding is consistent with the observation that APG-1387 also had only modest inhibitory effects on cell viability at 20 μM in these cell lines (Figures 2G,H). Similar results were found in Huh7 cells (Supplementary Figures 3a,b).

A previous report has shown that non-canonical nuclear factor-kappaB (NF-κB) signaling pathway activation resulted in tumor cell resistance to treatment with SMAC mimetics (Mak et al., 2014). The results of the present study showed that treatment with APG-1387 resulted in NF-κB inducing kinase (NIK) accumulation, and caused p100 processing to NF-κB2/p52 in a dose- and time-dependent manner, which further confirmed the activation of the non-canonical NF-κB pathway (Supplementary Figures 4a,b). Therefore, although APG-1387 monotherapy induced complete degradation of IAPs, treatment with APG-1387 had limited effect on cell apoptosis in HCC cell line HepG2 and HCCLM3 *in vitro*.

### APG-1387 Treatment Enhanced TNF-α- and TRAIL-Mediated Cell Killing Activities in HCC Cell Lines

**FIGURE 3:**
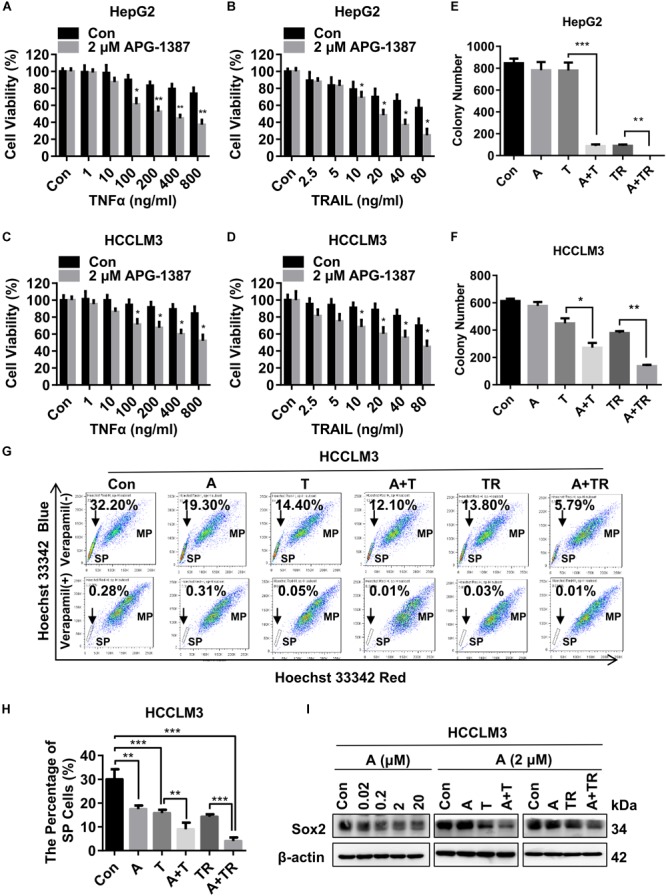
APG-1387 treatment enhanced TNF-α and TRAIL mediated anti-cancer activities in the HCC cell lines. **(A,B)** HepG2 and **(C,D)** HCCLM3 cells were pre-inoculated in quadruplicate at 2,000 cells per well in 96-well plates for 12 h, cell viability was evaluated using a CCK-8 assay after 24 h of stimulation with TNF-α or TRAIL, or in combination with APG-1387. **(E)** HepG2 and **(F)** HCCLM3 cells were pre-seeded in 6-well plates at 1,000 cells per well for 12 h and then stimulated with 2 μM APG-1387, 100 ng/ml TNF-α, 20 ng/ml TRAIL or their combination. After incubating for 14 days, the colonies were stained with Giemsa dye and counted with ImageJ software. **(G,H)** After 24 h of stimulation with APG-1387 and TNF-α or TRAIL, the percentages of side population (SP) cells were analyzed by flow cytometry after staining with Hoechst 33342 fluorescence dye alone or in combination with verapamil, which was used as a control to block the efflux of Hoechst 33342. **(I)** Under indicated treatment of APG-1387 and TNF-α or TRAIL, lysates of HepG2 and HCCLM3 cells were harvested, then the level of Sox2 protein was evaluated using a Western blot assay. Con, control; A, 2 μM APG-1387; T, 100 ng/ml TNF-α; TR, 20 ng/ml TRAIL; kDa, kilodalton. Error bars represented the mean ± SEM of triplicate representative experiments; ^∗^*p* < 0.05, ^∗∗^*p* < 0.01, ^∗∗∗^*p* < 0.001, by two-tailed unpaired *t*-test.

Both TNF-α and TRAIL are ligands that can trigger apoptosis in susceptible cancer cells by activating their corresponding cell death receptors (Fulda, 2015b). In present study, HepG2 and HCCLM3 cell lines were challenged with different concentrations of TNF-α or TRAIL, in the presence of APG-1387 (2 μM) and without APG-1387. Cell viability analysis using a cell counting kit-8 (CCK-8) and microscopic examination, showed that APG-1387 treatment enhanced TNF-α- or TRAIL-induced cell death in HepG2 cells (Figures 3A,B and Supplementary Figures 5a,b). Also, TNF-α- or TRAIL-induced cell death in HCCLM3 cells was enhanced by treatment with APG-1387 (Figures 3C,D and Supplementary Figures 5a,b). Furthermore, the findings showed that HepG2 and HCCLM3 cells treated with combination therapy, APG-1387 with TNF-α or TRAIL, showed increased inhibition of colony formation at 2 weeks when compared with monotherapy, which indicated that the combination of APG-1387 with TNF-α or TRAIL had long-term effects on the inhibition of HCC cell survival and proliferation (Figures 3E,F and Supplementary Figures 6a,b). Combination treatment using APG-1387 with TNF-α or TRAIL showed increased effects when compared with APG-1387 monotherapy in HCCLM3 cells, and the combination treatment achieved almost complete inhibition of colony formation in HepG2 cells. This disparity of anti-cancer activity by combination therapy between two cell lines may be due to the high frequency of CSCs in HCCLM3 cells (Figures 2A–C).

It has been reported that SP cells, which are typically identified based on their ability to efflux DNA-binding dye Hoechst 33342 through overexpressed ATP-binding cassette (ABC) transporters, such as ABCG2, have CSC-like characteristics and can be applied as a marker of CSCs for HCC (Chiba et al., 2006). In this study, the effects of APG-1387 treatment on CSC death were compared with TNF-α or TRAIL using the HCCLM3 cell line. Treatment with APG-1387 reduced the proportions of SP cells, with these effects being increased when used in combination with TRAIL (Figures 3G,H). The percentage of SP cells decreased from 32.20% in the untreated group to approximately 5.79% in the APG-1387 + TRAIL-treated group; the percentage of SP cells in the APG-1387, TNF-α, APG-1387 + TNF-α or TRAIL monotherapy groups was 19.30%, 14.40%, 12.10%, and 13.80%, respectively (Figures 3G,H). Also, the expression of the CSC marker Sox2 in HCCLM3 cells was reduced by APG-1387 as monotherapy or combination treatment (Figure 3I). These findings are consistent with previous findings in nasopharyngeal carcinoma cells *in vitro* (Wu et al., 2013; Ji et al., 2018). To examine whether the combination of APG-1387 with TNF-α or TRAIL specifically targets SP cells, we sorted SP and MP cells from the HCCLM3 cell line. Unfortunately, changes of cell viability indicated that SP cells were less sensitive to these combination therapies than MP cells (Supplementary Figures 7a,b).

### APG-1387 Treatment Increased TNF-α- and TRAIL-Induced Cell Death

**FIGURE 4:**
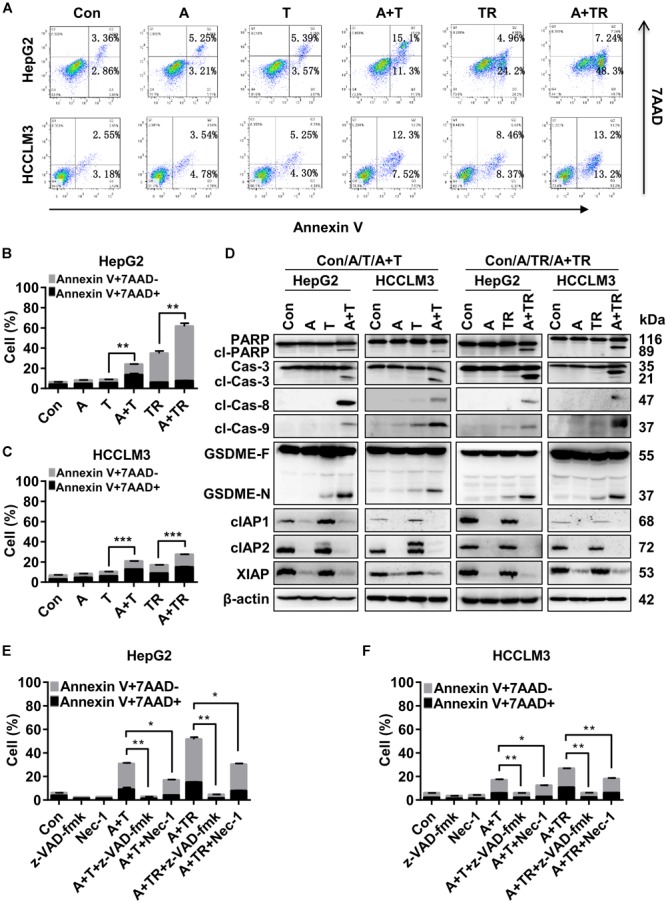
APG-1387 treatment enhanced TNF-α- and TRAIL-induced cell death in HepG2 and HCCLM3 cells. **(A–C)** HepG2 and HCCLM3 cells were seeded at 1 × 10^6^ cells per well in 6-well plates for 12 h. After 24 h of stimulation with APG-1387 and TNF-α or TRAIL, all of the cells were collected separately, and incubated with Annexin-V and 7-AAD for 15 min, then apoptosis (Annexin-V+ 7-AAD–) and necrosis (Annexin-V+ 7-AAD+) of HepG2 or HCCLM3 cells were detected by flow cytometry. **(D)** After 24 h of stimulation with APG-1387 and TNF-α or TRAIL, lysates of HepG2 and HCCLM3 cells were harvested. Western blot was performed to analyze the changes in levels of apoptosis-related proteins, including poly (ADP-ribose) polymerase (PARP), caspase-3, cleaved-caspase-8, cleaved-caspase-9, cIAP1, cIAP2, and XIAP and gasdermin E (GSDME). **(E,F)** HepG2 and HCCLM3 cells were pre-treated with a pan-caspase inhibitor (20 μM Z-VAD-fmk) or RIPK1 inhibitor (50 μM Nec-1) for 1 h. Then combination treatments involving APG-1387 with TNF-α or TRAIL were used, and the percentages of cell death were evaluated by flow cytometry. Con, control; A, 2 μM APG-1387; T, 100 ng/ml TNF-α; TR, 20 ng/ml TRAIL; cl-PARP, cleaved-PARP; Cas**-**3, caspase-3; cl-Cas**-**3, cleaved-caspase-3; cl-Cas**-**8, cleaved-caspase-8; cl-Cas**-**9, cleaved-caspase-9; GSDME-F, full-length GSDME; GSDME-N, N terminal fragment of GSDME; Nec-1, necrostatin-1; kDa, kilodalton. Error bars represented the mean ± SEM of triplicate representative experiments; ^∗^*p* < 0.05, ^∗∗^*p* < 0.01, ^∗∗∗^*p* < 0.001, by two-tailed unpaired *t*-test.

To determine the underlying mechanisms of synergistic cell death induced by APG-1387 combined with TNF-α or TRAIL, this study examined the effect of APG-1387 on TNF-α- and TRAIL-induced cell death in HepG2 and HCCLM3 cells. The flow cytometry assay with Annexin-V and 7-AAD staining, showed that APG-1387 was able to sensitize HepG2 cells to TNF-α- or TRAIL-induced apoptosis (Annexin V+ 7-AAD-) and necrosis (Annexin V+ 7-AAD+), while this effect was relatively weak in HCCLM3 cells (Figures 4A–C).

Additionally, Western blot analysis displayed that treatment with APG-1387 and TNF-α acted in concert to trigger the processing of caspase-3, -8, and -9 into the active cleavage fragments and resulted in the accumulation of cleaved PARP in both HepG2 and HCCLM3 cells, while the expression of IAPs were decreased (Figure 4D). Similar phenomena were observed in APG-1387 and TRAIL co-treated HepG2 and HCCLM3 cells (Figure 4D). It was reported that activation of caspase-3 cleaved gasdermin E (GSDME) during apoptosis, and the accumulation of the N-terminal fragment of GSDME resulted in secondary necrosis (Rogers et al., 2017; Zhang et al., 2018). In this study, when TNF-α or TRAIL combined with APG-1387 treatment, cleavage of GSDME was observed and the morphology of HepG2 and HCCLM3 cells showed increased bubble-like changes (Figure 4D and Supplementary Figures 5a,b), which were considered as signs of necrosis (Zhang et al., 2018). Consistently, induction of cell death in both cell lines by combination treatment was completely attenuated by the pan-caspase inhibitor, Z-VAD-FMK (Figures 4E,F and Supplementary Figure 8), and partially reduced by receptor-interacting serine/threonine-protein kinase 1 (RIPK1) inhibitor, necrostatin-1 (Nec-1) (Figures 4E,F and Supplementary Figure 8). These results indicated that APG-1387 enhanced TNF-α- or TRAIL-induced cell death via caspase-dependent apoptosis, and was partially dependent on RIPK1 kinase activity in human HCC cells *in vitro*.

### APG-1387 Sensitized HCC Cells to Natural Killer (NK) Cell-Mediated Cell Killing *in vitro* and *in vivo*

**FIGURE 5:**
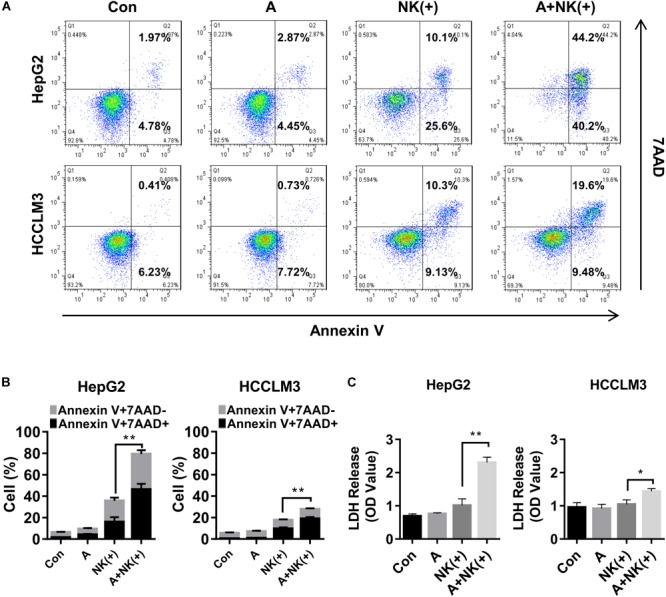
APG-1387 sensitized HepG2 and HCCLM3 cells to NK cell-mediated killing *in vitro.* HepG2 or HCCLM3 cells were co-cultured with purified NK cells, which were stimulated with 10 ng/ml interleukin (IL)-12, 10 ng/ml IL-15, and 100 ng/ml IL-18, either alone or in the presence of 2 μM APG-1387. **(A,B)** After co-incubation for 24 h, HepG2 or HCCLM3 cells were collected for analysis of killing effect of NK cells by flow cytometry, **(C)** while supernatants were collected for the LDH assay. Con, control; A, 2 μM APG-1387; NK(+), NK cells co-cultured with 10 ng/ml IL-12, 10 ng/ml IL-15, and 100 ng/ml IL-18. Error bars represented the mean ± SEM of triplicate representative experiments; ^∗^*p* < 0.05, ^∗∗^*p* < 0.01; by paired *t*-test.

Natural killer cells are a key component of the innate immune response to tumors, including to HCC, and they have recently been studied as potential therapeutic agents in HCC (Sun et al., 2015). Therefore, the tumor cell killing effect of NK cells combined with APG-1387 was investigated on HepG2 and HCCLM3 cells. The results showed that the proportions of apoptotic and necrotic cells among HepG2 and HCCLM3 cells were significantly increased when they were treated with pre-activated NK cells combined with 2 μM of APG-1387 (Figures 5A,B). Similar results were obtained when LDH was measured, which was used as an enzyme marker of the degree of cell death (Figure 5C). Interestingly, APG-1387 treatment had no significant cytotoxic effect on NK cells but resulted in a slight increase in TNF-α and interferon-gamma (IFN-γ) expression in pre-activated NK cells and supernatant (Supplementary Figures 9a–c).

**FIGURE 6:**
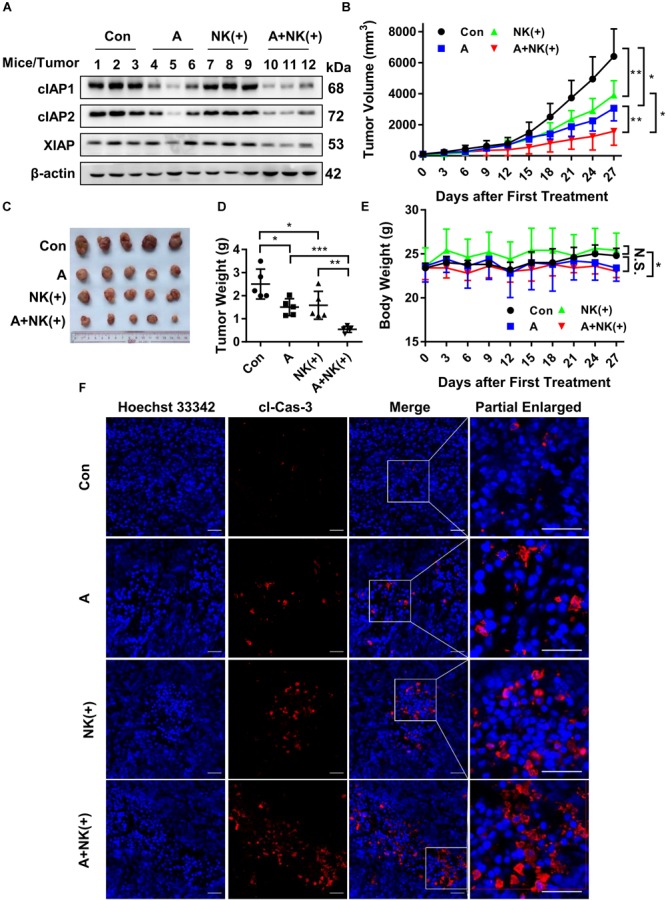
APG-1387 sensitized HCCLM3 tumors toward NK cell-mediated killing *in vivo.* Four groups of NOD-SCID mice bearing human HCC cell line HCCLM3 tumors were injected intraperitoneally with APG-1387 at 20 mg/kg every 3 days for 4 weeks, and/or injected at the tumor site with 2 × 10^7^ IL-12, IL-15, and IL-18-activated NK cells per mouse (or with the same volume of PBS) every 6 days for 4 weeks. **(B,E)** Tumor volume and body weight were measured every 3 days. **(C,D)** After 4 weeks of treatment, the mice were sacrificed, the tumors were harvested and weighed. **(A)** The expression of IAPs in tumors was measured by Western blot and **(F)** the relative proportion of cleaved caspase-3 was shown using confocal microscopy. Con, control; A, APG-1387; NK(+), NK cells co-cultured with 10 ng/ml IL-12, 10 ng/ml IL-15, and 100 ng/ml IL-18; cl-Cas-3, cleaved-caspase-3. ^∗^*p* < 0.05, ^∗∗^*p* < 0.01, ^∗∗∗^*p* < 0.001, N.S., not significant; by two-tailed unpaired *t*-test.

Finally, the efficacy of APG-1387 and pre-activated NK cells was studied in an HCCLM3 xenograft in non-obese diabetic and severe combined immunodeficiency (NOD-SCID) mice, which had impaired T, B and NK cell function. The findings were that APG-1387 administration decreased the expression of cIAP1 and cIAP2, and less potent to XIAP expression (Figure 6A). Moreover, APG-1387 potentiated the effects of pre-activated NK cells on HCCLM3 xenograft tumor growth and tumor weight (Figures 6B–D). Also, confocal immunofluorescence microscopy showed that the combination of APG-1387 and pre-activated NK cell treatment resulted in the highest ratio of cleaved caspase-3-positive cells in harvested tumors (Figure 6F). In the NOD-SCID mouse HCCLM3 xenograft tumors, APG-1387 monotherapy also exhibited some degree of anti-tumor effect (Figures 6B–D), despite being shown to be ineffective *in vitro*, as a single agent. Also, even though the body weight of mice decreased following APG-1387 administration, this weight loss did not continue and eventually stabilized, indicating that APG-1387 was well tolerated (Figure 6E).

## Discussion

Indeed, the expression of IAPs between HCC and adjacent normal liver tissue has been previously reported (Augello et al., 2009). They found cIAP1, cIAP2, and XIAP was not significantly increased in HCC, which was inconsistent with our results. This difference might be due to the heterogeneity of tumor tissues. Although IAPs are involved in the regulation of innate immune (Estornes and Bertrand, 2015) and HCC are often accompanied by immune cell infiltration, we found cIAP1 and cIAP2 were exclusively expressed in the cytoplasm in both neoplastic and non-neoplastic hepatocytes, which was similar to the expression of XIAP (Augello et al., 2009). The protein levels of cIAP1 and cIAP2 degraded rapidly in HCC cell lines in the presence of APG-1387. Although HepG2 and HCCLM3 cells were not as sensitive to the APG-1387-induced killing as reported in PLC/PRF/5 cells (Pan et al., 2018), they became susceptible to apoptosis induced by TNF-α and TRAIL when co-incubated with APG-1387. Moreover, APG-1387 might also enhance the tumor inhibitory effect of TNF-α and TRAIL in HCC CSCs. The data from this study also indicated that APG-1387 treatment enhanced the killing effect of NK cells on HCC cells *in vitro* and *in vivo*, while increasing the cytotoxic potential of NK cells.

Defects in programmed cell death, or apoptosis, including abnormal expression of proteins encoded by the *IAP* gene, have been documented in several types of cancer (Fulda and Vucic, 2012). Overexpression of *IAPs* usually implies poor prognosis in patients with cancer (Fulda and Vucic, 2012). The results of the present study showed that the mRNA and protein levels of *cIAP1, cIAP2*, and *XIAP* were increased in HCC tissues compared with adjacent normal liver tissues. Therefore, IAPs may have a role in HCC development and might be promising targets for HCC treatment. However, further studies are required to confirm these findings and to determine the mechanisms of action of IAPs in HCC.

There are several approaches to down-regulate the levels of IAPs, including the use of SMAC-based peptides, IAP-antisense oligonucleotides, and SMAC mimetics (Vucic and Fairbrother, 2007; Fulda and Vucic, 2012). Among of these strategies, the SMAC mimetics may represent the most effective strategy for antagonizing endogenous IAP proteins (Vucic and Fairbrother, 2007; Fulda and Vucic, 2012). When compared with monovalent SMAC mimetics, bivalent SMAC mimetics display higher antitumor activity and more effective degradation of IAPs, probably because they are more effective in blocking XIAP-mediated caspase inhibition (Sun et al., 2007). In the present study, the bivalent SMAC mimetic APG-1387 was able to induce almost complete reduction of cIAP1 and cIAP2, and suppressed XIAP to some extent in HCC cell line HepG2 and HCCLM3. However, unlike the significant anti-tumor effects of APG-1387 on ovarian cancer (Li et al., 2018), nasopharyngeal carcinoma (Li et al., 2016) and PLC/PRF/5 cells (Pan et al., 2018), APG-1387 treatment alone, only showed limited inhibitory effects on HCC cell lines HepG2, HCCLM3 and Huh7, suggesting that HCC is more resistant to the SMAC mimetic than ovarian cancer or nasopharyngeal carcinoma.

Despite some signs of the antitumor effects of SMAC mimetics as single agents, most types of malignancy are not sensitive to their ability to induce tumor cell death. Certain types of malignant cells can develop resistance to the effects of cytokines, due to the accumulation of IAPs (Varfolomeev et al., 2008; Petersen et al., 2010). However, SMAC mimetics can cause significant effects in certain cancer cells because they are capable of producing a large quantity of apoptosis-inducing proteins, including inflammatory cytokines. For example, PLC/PRF/5 cells express high level of TNF-α and are sensitive to APG-1387 single agent (Pan et al., 2018). Previous studies have shown that the inhibition of IAPs by SMAC mimetics can contribute to TNF-α-induced cell death (Wu et al., 2013; Fulda, 2015a; Li et al., 2018). In the present study, APG-1387 enhanced the cytotoxic effects of TNF-α and TRAIL on HepG2 and HCCLM3 cells to some extent, but the combination of APG-1387 with TNF-α or TRAIL resulted in a strong inhibitory effect on clonogenic survival, demonstrating long-term anti-tumor activity. The underlying mechanism of these combination treatments was likely to be due to the enhanced activation of caspase-8, -9, and -3, and the induction of apoptosis. The cytotoxicity of APG-1387 combined with TNF-α or TRAIL, could be completely blocked by the pan-caspase inhibitor Z-VAD-FMK, and partially attenuated by the RIPK1 inhibitor Nec-1, indicating that the combination treatments were caspase-dependent and partially RIPK1-dependent. This finding is supported by studies that have demonstrated the use of SMAC mimetics in combination with TNF-α, TRAIL, IFN-γ, or certain methods of producing inflammatory cytokines to exploit synergistic effects on tumor cell death (Fulda and Vucic, 2012; Beug et al., 2014; Tanzer et al., 2017).

Natural killer cells constitute a major component of the innate immune system against tumors. NK cells are enriched in healthy livers and are believed to have an important role in immune surveillance against abnormal or neoplastic liver cells (Sun et al., 2015). The number of NK cells in blood and tumor tissues has been previously shown to be positively correlated with the survival and prognosis of patients with HCC (Cai et al., 2008). However, dysfunction and depletion of NK cells had been reported in patients with advanced-stage HCC (Cai et al., 2008). Previously, Pan et al. (2018) showed that APG-1387 promoted the proliferation of NK cells in PBMCs and increased the number of NK cells in PLC/PRF/5 tumor xenograft, however, the influence of APG-1387 on the function of NK cells and the combinational effect on HCC remained to be explored. In the present study, the findings showed that the cytotoxic effect of NK cells on HCC cells was improved by the use of the SMAC mimetic, APG-1387, *in vitro* and *in vivo*, suggesting that NK cell-based immunotherapy combined with SMAC mimetic treatment might be a promising future anti-tumor strategy.

Currently, the long-term prognosis of HCC has improved due to the emergence of improved clinical diagnostic and therapeutic strategies, but there are still some drawbacks associated with these treatments, including their inability to eliminate CSCs, which may explain treatment resistance in some patients with HCC. Therefore, selective targeted therapy for HCC CSCs might result in more effective treatments. It has been previously reported that IAPs are frequently overexpressed in CSCs of certain malignancies, such as glioblastoma and nasopharyngeal carcinoma (Liu et al., 2006; Wu et al., 2013). In the present study, the expression of IAPs and CSC-related genes and the frequency of SP cells were higher in HCCLM3 cells than HepG2 cells. SP cells showed increased expression of IAPs when compared with the rest of the MP cells in the HCCLM3 cell line. Therefore, antagonism of IAPs appears to be a promising method of targeting HCC CSCs. APG-1387 in combination with TRAIL was shown to selectively target CSCs and down-regulate CSC properties of HCC in HCCLM3 cells, which is consistent with the previous findings in glioblastoma and nasopharyngeal carcinoma (Wu et al., 2013; Tchoghandjian et al., 2014).

## Conclusion

In conclusion, although HCC cell line HepG2 and HCCLM3 were resistant to APG-1387 monotherapy, APG-1387 could sensitize them to TNF-α- or TRAIL-induced apoptosis, CSC inhibition, and also potentiated the cytotoxic effects of NK cells on these HCC cell lines *in vitro* and *in vivo*. The findings of this study suggest that SMAC mimetics, such as APG-1387, should be studied further as potential therapeutic agents in HCC, and this might offer a new approach for the therapy of chemotherapy-resistant HCC, particularly when used in combination with immunotherapy agents.

## Availability of Data and Material

All data generated or analyzed during this study are available from the corresponding author on reasonable request.

## Author Contributions

XZ conceived and designed the study. ZC, JC, HL, WD, and XH performed the experiments and analyzed the data. XZ and ZC wrote the manuscript with additional input and suggestions from DY and JH. All authors reviewed and approved the manuscript.

## Conflict of Interest Statement

DY is a co-founder and the chief executive officer of Ascentage Pharma Group Inc. The remaining authors declare that the research was conducted in the absence of any commercial or financial relationships that could be construed as a potential conflict of interest.

## Footnotes

1 https://cancergenome.nih.gov
