# Supplementary material for: The SMAC Mimetic APG-1387 Sensitizes Immune-Mediated Cell Apoptosis in Hepatocellular Carcinoma
Source: Front Pharmacol. 2018 Nov 6;9:1298. doi: 10.3389/fphar.2018.01298 (PMC6232623; doi:10.3389/fphar.2018.01298)
Supplement: Supplementary file 1 [file Data_Sheet_1.pdf]

Supplementary Material

SMAC mimetic APG-1387 targets the inhibitor of apoptosis proteins and sensitizes immune-mediated cell apoptosis in hepatocellular carcinoma

Zide Chen[1], Jiehua Chen[1], Hongyan Liu[1], Wei Dong[2], Xuan Huang[1], Dajun Yang[3], Jinlin Hou[1], Xiaoyong Zhang[1, *]

*** Correspondence:** Xiaoyong Zhang: xiaoyzhang@smu.edu.cn

Supplementary Figures and Tables

1 Supplementary Figures

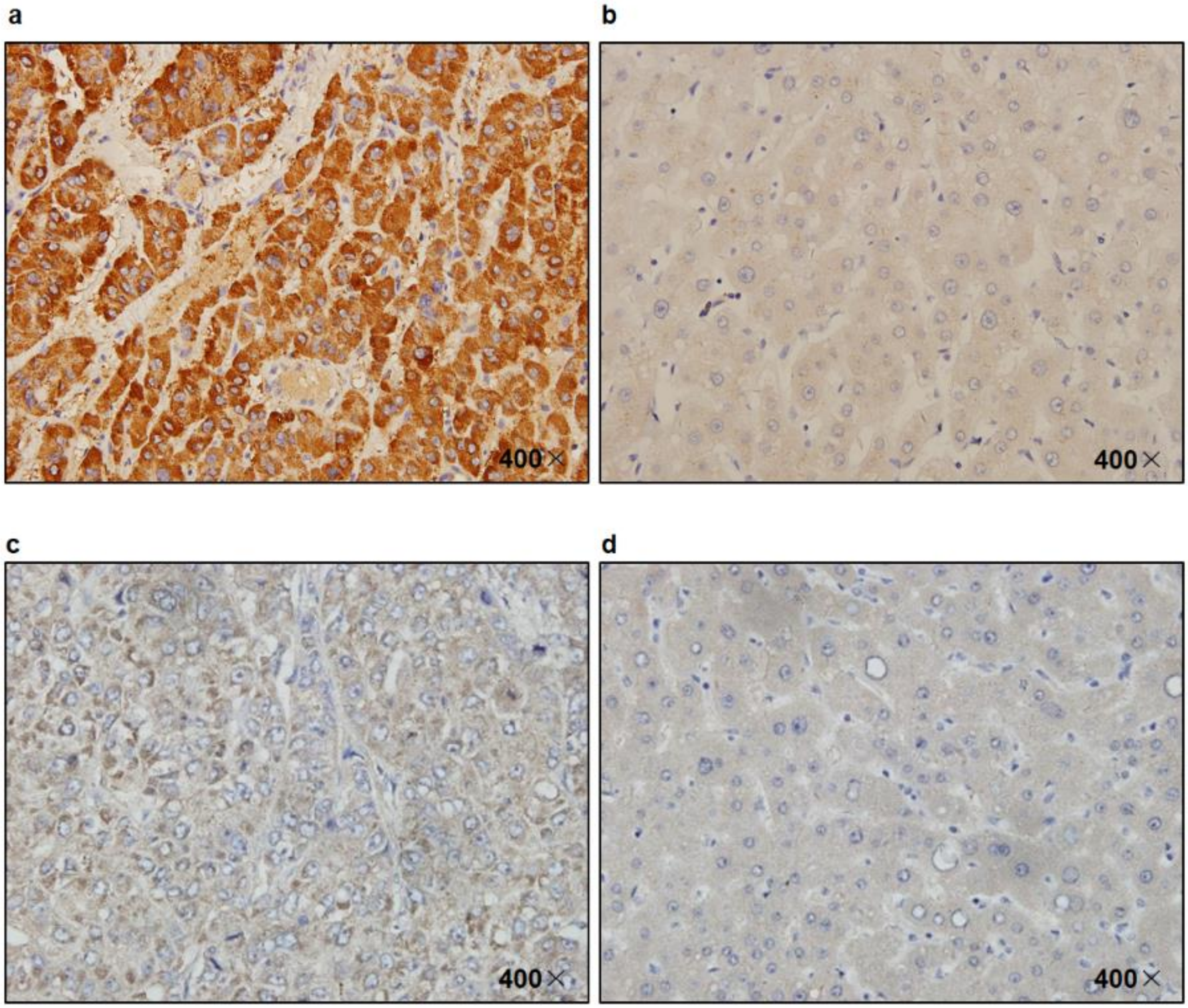

Supplementary Figure 1. The localization of cIAP1 and cIAP2 proteins in HCC tumor tissue. Immunohistochemical analysis of paraffin-embedded HCC (**a, c**) and adjacent normal liver tissue (**b, d**) showed that cIAP1 (**a, b**) and cIAP2 (**c, d**) were mainly located in the cytoplasm of hepatoma cells and normal hepatocytes. 400 × , 400 × magnification.

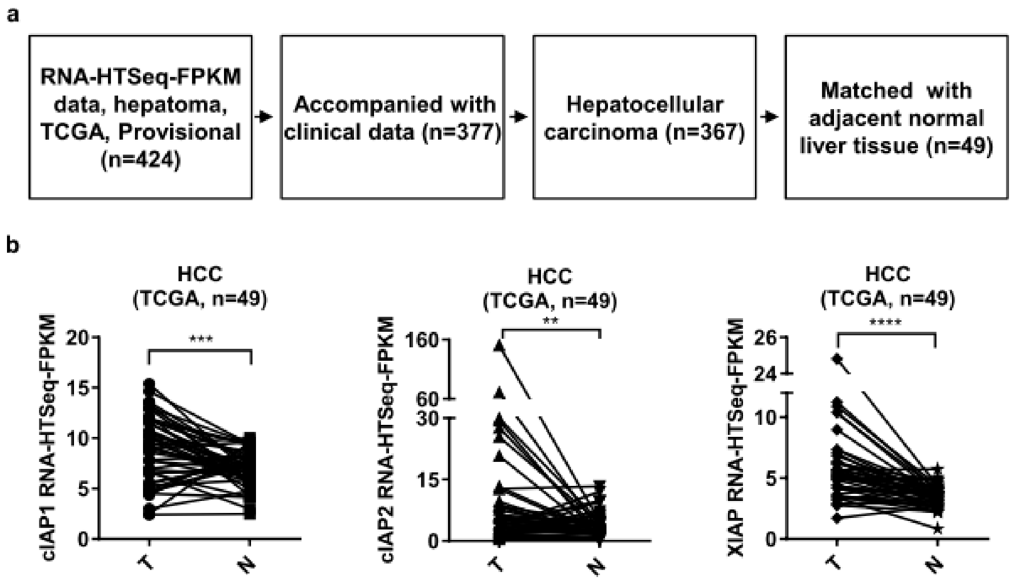

Supplementary Figure 2. Inhibitor of apoptosis protein (*IAP*) genes were highly expressed in HCC tissue. (**a**) Screening strategy of HCC patients from the cancer genome atlas (TCGA) database (updated until Jun 02, 2016; http://tcga-data.nci.nih.gov/). (**b**) The expressions of *cIAP1*, *cIAP2*, and *XIAP* mRNA in tumor and normal adjacent liver tissue from 49 HCCs were compared. RNA-HTSeq-FPKM, RNA-high throughput sequencing-fragments per kilobase of exon per million mapped reads; T, HCC tumor tissue; N, normal adjacent liver tissue. ** $p < 0.01$, *** $p < 0.001$, *** $p < 0.0001$, by two-tailed pair t-test.

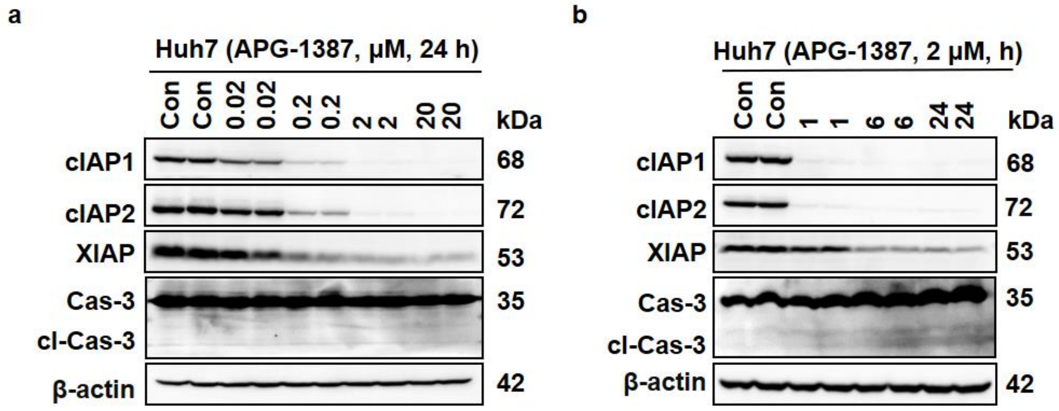

Supplementary Figure 3. The influence of APG-1387 on IAPs and caspase-3 in HCC cell line, Huh7, as a single drug. (**a, b**) Huh7 cells were seeded at 1.5×10^6 cells per well in 6-well plates for 12 hours. After 24 hours of stimulation with 0, 0.02, 0.2, 2, 20 μM APG-1387 respectively, or 0, 1, 6, 24 hours stimulation of 2 μM APG-1387, lysates of Huh7 cells were collected and analyzed by Western blot to show the changes in cIAP1, cIAP2, XIAP and caspase-3 protein levels. Con, control; Cas-3, caspase-3; kDa, kilodalton.

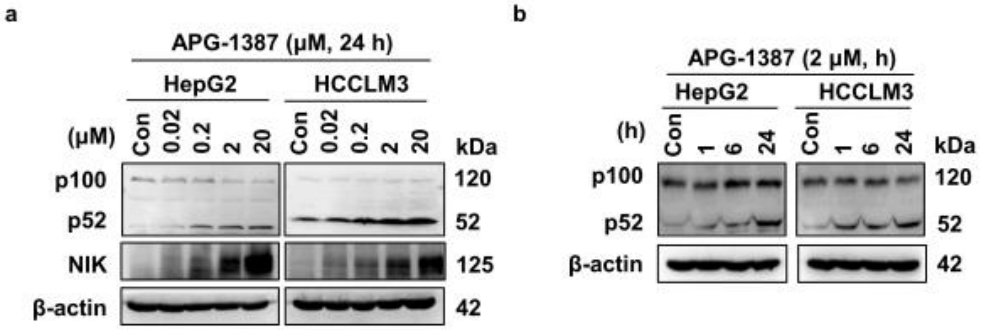

Supplementary Figure 4. APG-1387 activated non-classical NF-κB signaling of HCC cell lines. HepG2 and HCCLM3 cells were seeded at 1.5×10^6 cells per well in 6-well plates for 12 hours. (**a, b**) After stimulating with different concentrations of APG-1387 for specified times, lysates of HepG2 and HCCLM3 cells were collected. The protein levels of p100, p52 and NF-κB inducing kinase (NIK) were analyzed by Western blot. Con, control; kDa, kilodalton.

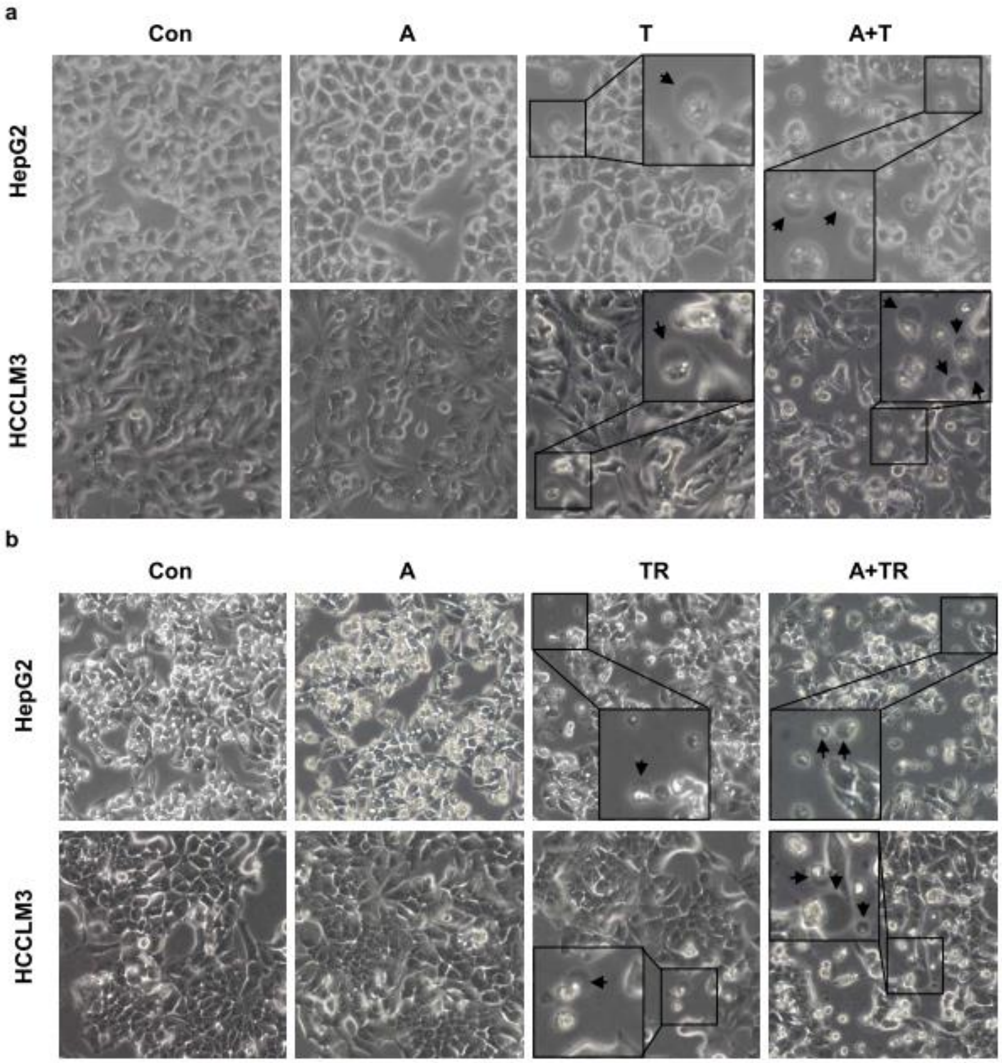

Supplementary Figure 5. The morphology changes of HCC cells, HepG2 and HCCLM3, after stimulation with APG-1387 and TNF-α or TRAIL. (**a**) HepG2 and (**b**) HCCLM3 cells were seeded at 1×10^6 cells per well in 6-well plates for 12 hours. After 24 hours of stimulation with APG-1387 and TNF-α or TRAIL, the morphologic alterations of HepG2 or HCCLM3 were captured under inverted optical microscope (magnification, 100×). The arrows in the enlarged part (magnification, 400×) of the picture showed the images of cell necrosis. Con, control; A, 2 μM APG-1387; T, 100 ng/ml TNFα; TR, 20 ng/ml TRAIL.

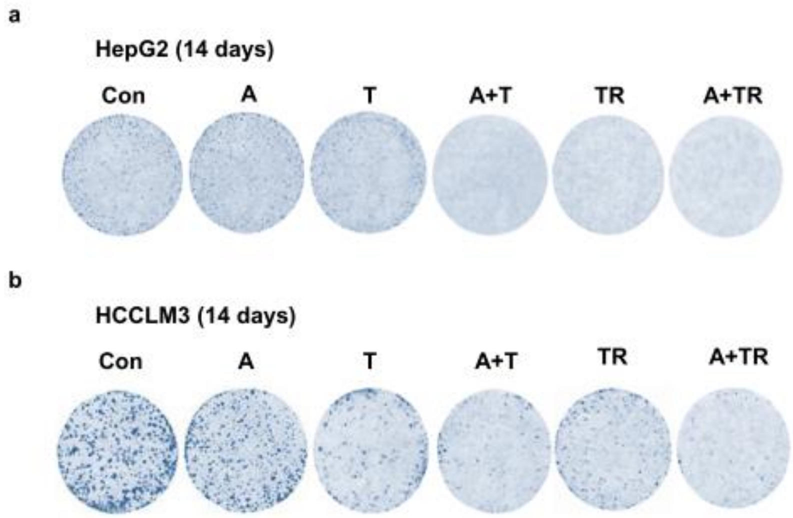

Supplementary Figure 6. APG-1387 treatment enhanced the inhibitory effect of TNF-α or TRAIL on the proliferation of HCC cells. (**a**) HepG2 and (**b**) HCCLM3 cells were pre-seeded in 6-well plates at 1,000 cells per well for 12 hours and then stimulated with 2 μM APG-1387, 100 ng/ml TNF-α, 20 ng/ml TRAIL or their combination. After incubating for 14 days, the colonies were stained with Giemsa dye and counted with Image J software.

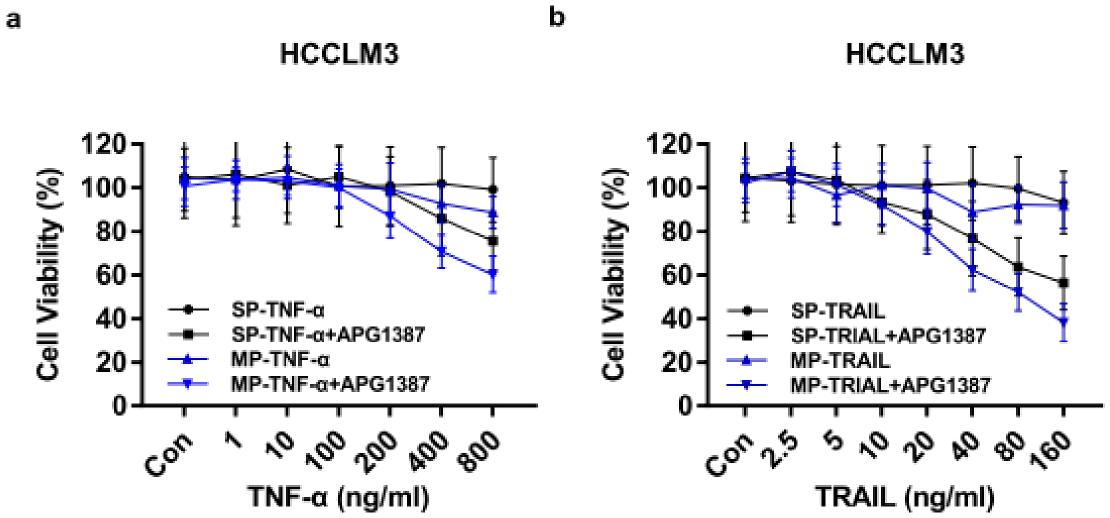

Supplementary Figure 7. The susceptibility of side population (SP) and major population (MP) cells in HCCLM3 cell line to the combination of APG-1387 and TNF-α/TRAIL. SP and MP cells were sorted from HCCLM3 cell line by flow cytometry and pre-inoculated in triplicate at 2,000 cells per well in 96-well plates for 12 hours. Cell viabilities were evaluated using a CCK-8 assay after 24 hours' stimulation with TNF-α (**a**) or TRAIL (**b**), or in combination with APG-1387. Con, control.

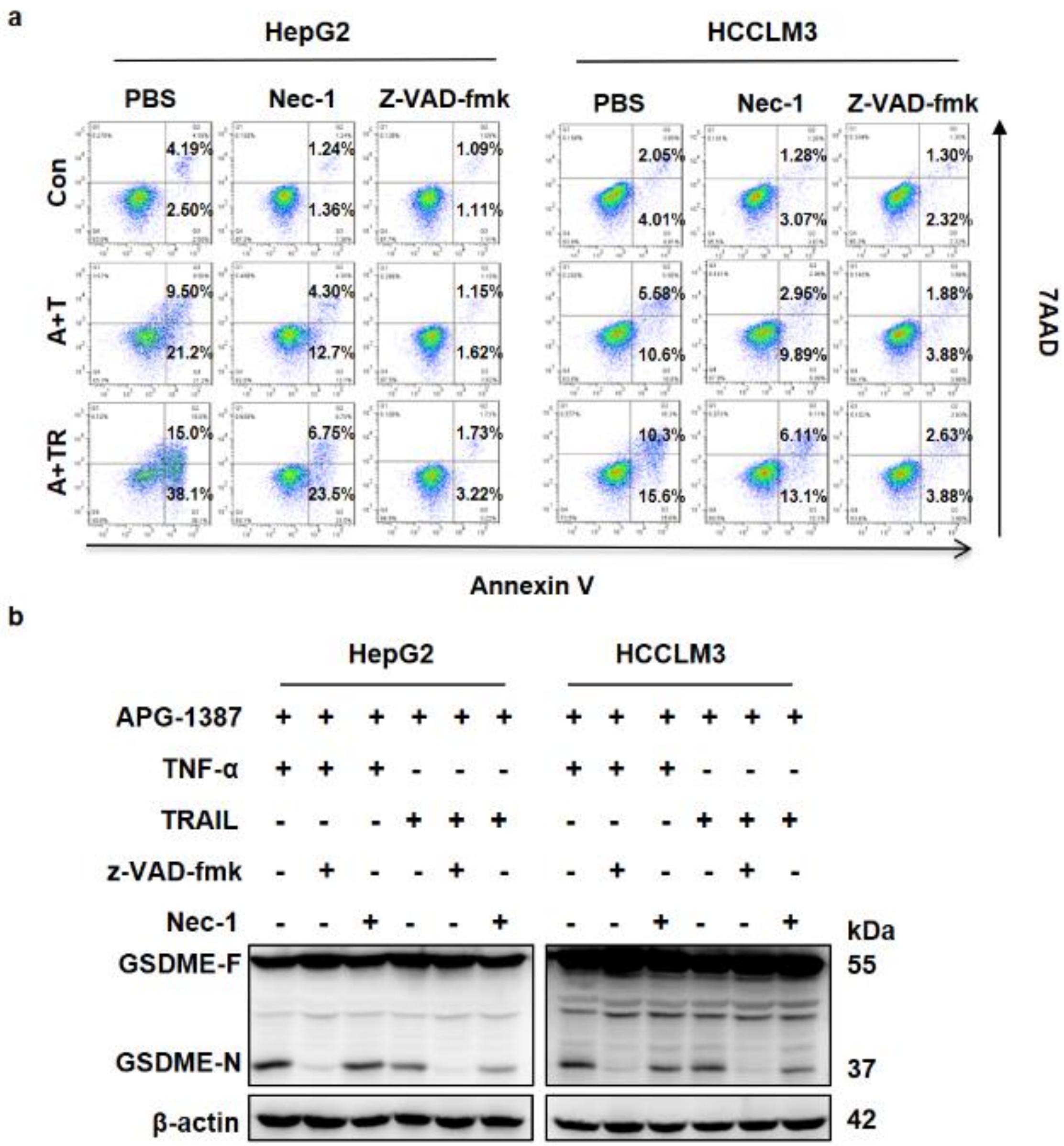

Supplementary Figure 8. APG-1387 treatment enhanced TNF-α- and TRAIL-induced cell death in HepG2 and HCCLM3 cells. HepG2 and HCCLM3 cells were pre-treated with a pan-caspase inhibitor (20 μM Z-VAD-fmk) or RIPK1 inhibitor (50 μM Nec-1) for 1 hour. Then the combination treatments involving 2 μM APG-1387 with 100 ng/ml TNF-α or 20 ng/ml TRAIL were used. Cell death were assessed by flow cytometry (**a**) and Western blot (**b**). Nec-1, necrostatin-1; GSDME-F, full-length GSDME; GSDME-N, N terminal fragment of GSDME.

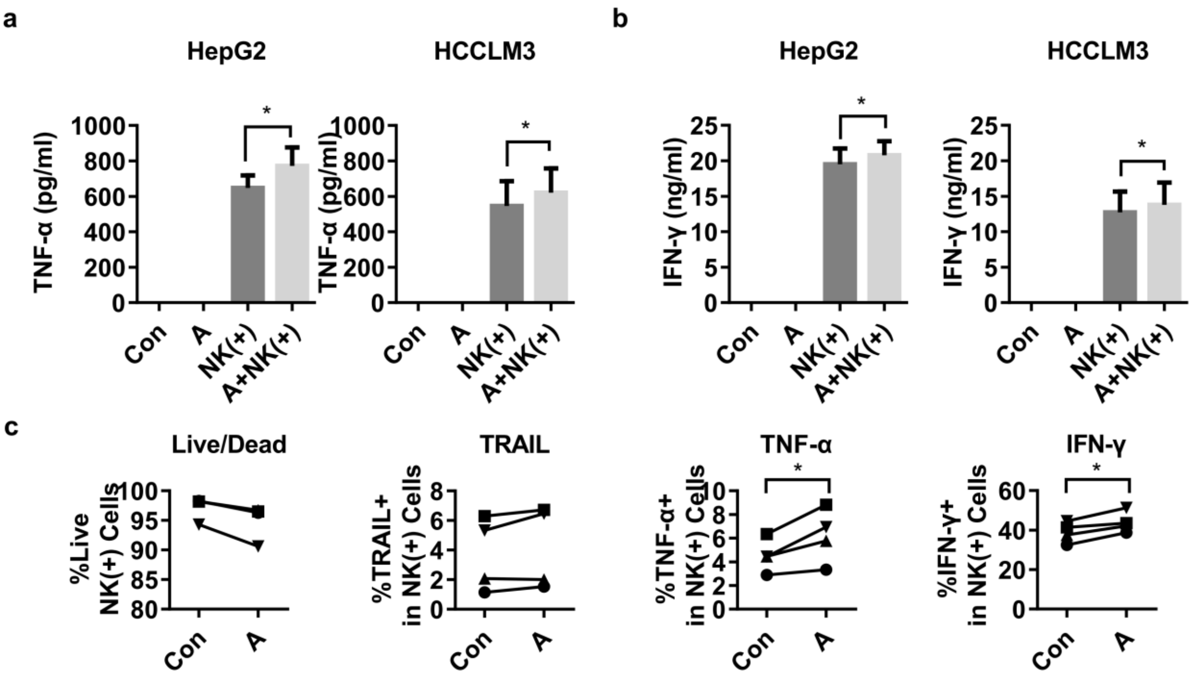

Supplementary Figure 9. Changes in cytotoxic potential of interleukin (IL)-12, IL-15, and IL-18 activated NK cells after APG-1387 stimulation. (**a, b**) HepG2 or HCCLM3 cells were co-cultured with purified NK cells, which stimulated with 10 ng/ml IL-12, 10 ng/ml IL-15 and 100 ng/ml IL-18, either alone or in the presence of 2 μM APG-1387. After co-incubation for 24 hours, supernatants were collected for the examination of TNF-α and IFN-γ by ELISA. (**c**) NK cells stimulated with 10 ng/ml IL-12, 10 ng/ml IL-15 and 100 ng/ml IL-18, were co-cultured with 2 μM APG-1387 or not for 24 hours, then the percentage of living cells, TRAIL expression, TNF-α and IFN-γ in NK cells were analyzed by flow cytometry. Con, control; A, 2 μM APG-1387; NK(+), NK cells co-cultured with 10 ng/ml IL-12, 10 ng/ml IL-15 and 100 ng/ml IL-18; Live/Dead, a membrane and intracellular dye (ThermoFisher, cat. no. L34976) that can distinguish live cells from stained dead cells. Error bars represented the mean ± S.E.M. of triplicate representative experiments; * $P < 0.05$, by two-tailed paired t-test.

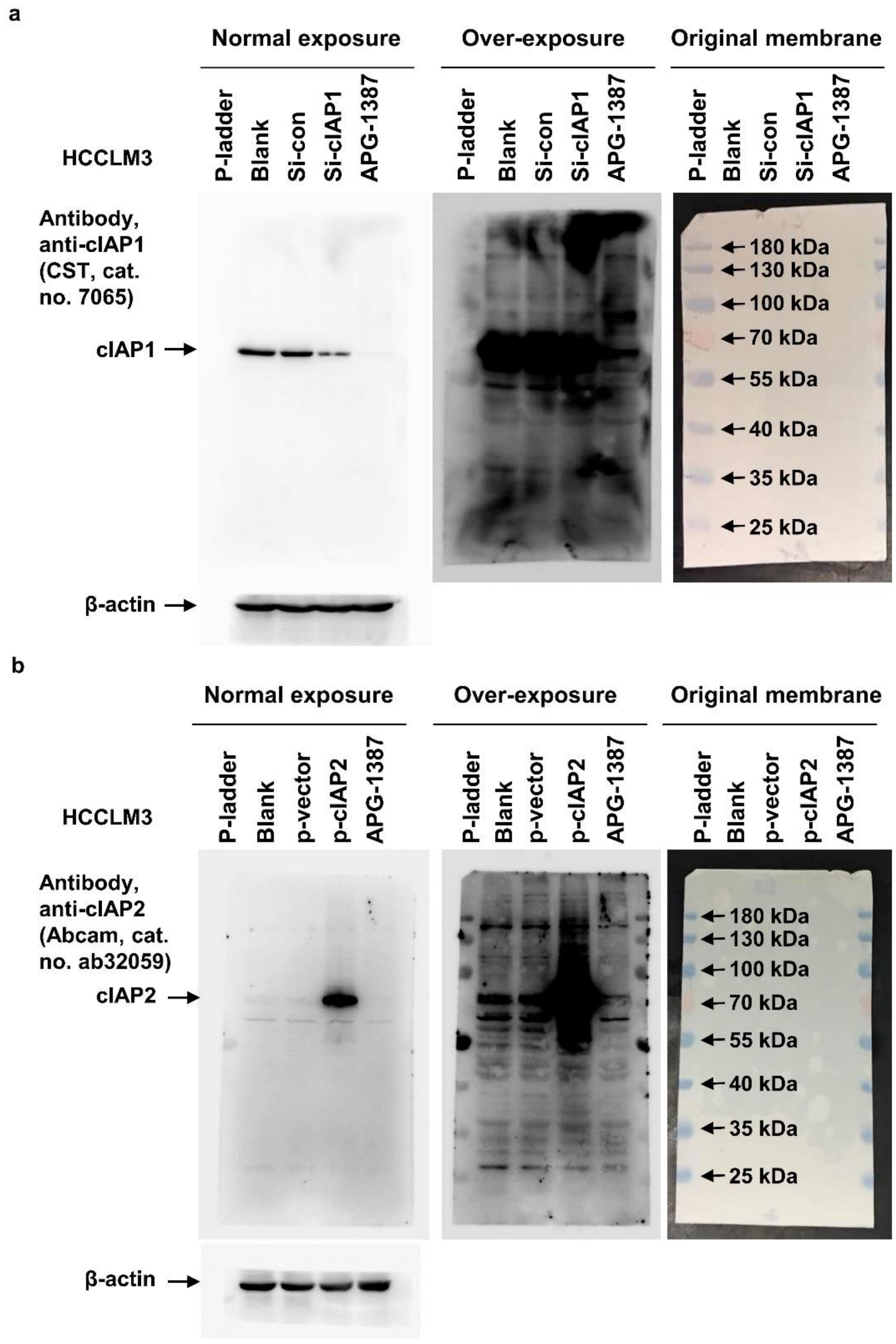

Supplementary Figure 10. Validation of cIAP1 and cIAP2 antibody. (**a**) For cIAP1 antibody, HCCLM3 cells were transfected with cIAP1 small interfering RNA (si-cIAP1), control small interfering RNA (si-con) or 2 µM APG-1387; (**b**) for cIAP2 antibody, HCCLM3 cells were transfected with cIAP2 plasmid (p-cIAP2), negative control plasmid vector (p-vector) or 2 µM APG-1387. After 48 hours treatment, cell lysates were collected. Western blot analysis was used to detect specificity of cIAP1 antibody (CST, cat. no. 7065) and cIAP2 antibody (Abcam, cat. no. ab32059). P-ladder, Pre-stained protein ladder (Life, cat. no. 26616); APG-1387, 2 µM APG-1387.

2 Supplementary Tables

Supplementary Table 1. Clinical characteristics of 12 HCC patients from Tongji hospital

Variable	Number/Mean±SD (range)
Age (year)	53.17 ± 3.657, (26-68)
Gender (male or female)	10/2
HBV (+/-)	12/0
HBsAg (>250/≤250 IU/mL)	9/3
HBsAb (>10/≤10 IU/L)	2/10
HBeAg (>1/≤1 S/CO)	4/8
HBeAb (>1/≤1 S/CO)	5/7
HBcAb (>1/≤1 S/CO)	12/0
HCV (+/-)	0/12
History of Schistosoma (+/-)	0/12
History of smoking (+/-)	4/8
History of drinking (+/-)	3/9
diabetes mellitus (+/-)	7/5
Hepatic encephalopathy (+/-)	1/11
Ascites (+/-)	4/8
Pathological grading (poorly/moderately/well differentiation)	4/4/4
Number of tumor nodules (>1/≤1)	2/10

Tumor size (>3/≤3 cm)	11/1
Portal vein tumor thrombus (+/-)	4/8
Distance metastasis (+/-)	1/11
Tumor encapsulation (+/-)	6/6
Cirrhosis (+/-)	10/2
Alanine aminotransferase (≥40/＜40 U/L)	3/9
alpha-Fetoproteins (≥20/＜20 μg/L)	8/4

SD, standard deviation;

HBV, hepatitis B virus; HBsAg, HBV surface antigen; HBsAb, antibody to HBsAg; HBeAg, HBV e Antigens; HBeAb, antibody to HBeAg; HBcAb, HBV core antibody; HCV, hepatitis C virus.

Supplementary Table 2. HCC patient characteristics data from TCGA public database

**Characteristics**	**HCC matched with adjacent normal liver tissue (n=49)**
Age (year)	61.4±16.2
(range)	(20-81)
Gender (male/female)	28/21
TNM	
T1/T2/T3/T4	20/13/13/3
N0/N1/Nx	31/1/17
M0/M1/Mx	32/1/16
Stage (I/II/III/IV/-)	18/10/12/1/8
Pathological grade (1/2/3/-)	5/26/15/3

HCC, hepatocellular carcinoma;

T, local tumor; N, lymph node; M, metastases.
